# Probucol Attenuates Oxidative Stress, Energy Starvation, and Nitric Acid Production Following Transient Forebrain Ischemia in the Rat Hippocampus

**DOI:** 10.1155/2011/471590

**Published:** 2011-08-04

**Authors:** Abdulhakeem A. Al-Majed

**Affiliations:** Department of Pharmacology and Toxicology, College of Pharmacy, King Saud University, P.O. Box 2457, Riyadh 11451, Saudi Arabia

## Abstract

Oxidative stress and energy depletion are believed to participate in hippocampal neuronal damage after forebrain ischemia. This study has been initiated to investigate the potential neuroprotective effects of probucol, a lipid-lowering drug with strong antioxidant properties, against transient forebrain ischemia-induced neuronal damage and biochemical abnormalities in rat hippocampal CA1 region. Adult male Wistar albino rats were subjected to forebrain ischemia and injected with probucol for the next 7 successive days, and compared to controls. Forebrain ischemia resulted in a significant decrease in the number of intact neurons (77%), glutathione (GSH), and adenosine triphosphate (ATP), and a significant increase in thiobarbituric acid reactive substances (TBARS) and total nitrate/nitrite, (NO_*x*_) production in hippocampal tissues. The administration of probucol attenuated forebrain ischemia-induced neuronal damage, manifested as a complete reversal of the decrease in the number of intact neurons, ATP and GSH and the increase in TBARS and NO_*x*_ in hippocampal tissues. This study demonstrates that probucol treatment abates forebrain ischemia-induced hippocampal neuronal loss, energy depletion, and oxidative stress in hippocampal CA1 region. Thus, probucol could be a promising neuroprotective agent in the treatment of forebrain ischemia.

## 1. Introduction

Damage to brain tissue due to ischemic stroke is the first leading cause of fatal neurological disease, the third cause of death after heart disease and cancer, and is the major cause of adult long-term disability in industrialized countries [1–3]. A significant number of strokes is a consequence of the occlusion of one of the main or secondary cerebral arteries [4, 5]. Transient global ischemia (forebrain ischemia), occurring during cardio-respiratory arrest in patients or in experimental animals, induces selective and delayed neuronal cell death. The hippocampus, particularly the CA1 pyramidal neurons, is the most severely affected brain structure by global ischemia [2, 5]. The damage to the hippocampal CA1 sector develops 3–7 days after untreated forebrain ischemia in rats, gerbil, and human [2, 6]. Forebrain ischemia is caused by a deficiency in the blood supply to hippocampal neurons, leading to disturbances in energy metabolism that lead to a decrease in glucose utilization, with a consequent decrease in the production of ATP and phosphocreatine [7, 8]. This leads to anaerobic glycolysis and lactic acidosis followed by membrane depolarization, influx of calcium ions, and the release of glutamate into the extracellular space that induces pro-oxidant enzymes such as inducible nitric oxide synthase (iNOS) [9, 10]. Most of these changes are associated with a massive production of reactive oxygen species (ROS), which causes severe oxidative injury to the brain tissue [11]. Also, reperfusion is associated with a massive production of toxic ROS that potentiates the initial brain damage caused by forebrain ischemia [12]. The accumulation of the toxic ROS markedly increases the susceptibility of brain tissues to oxidative damage via membrane lipid peroxidation, protein, and DNA oxidation [11–13]. Earlier studies have reported that oxidative damage is a primary factor in various models of acute brain damage including forebrain ischemia [13–15]. Moreover, increased oxidative stress biomarkers and depletion of enzymatic and nonenzymatic antioxidants have been reported in many diseases including forebrain ischemia-induced neuronal damage [16, 17]. Thus, one strategy to protect the brain against forebrain ischemia-induced hippocampal neuronal damage is to decrease oxidative damage by neutralizing the toxic ROS that are produced in the ischemic tissues.

Probucol is a clinically used cholesterol-lowering drug with pronounced antioxidant effect [18]. Besides its antioxidant properties, probucol was shown to protect against diabetes and doxorubicin-induced cardiomyopathy associated with oxidative stress by stimulating the endogenous antioxidant enzymes [19–21]. Previous studies have demonstrated that probucol improved survival of rats with large myocardial infarction by reducing cardiac fibrosis and expression of inflammatory cytokines [22, 23]. In doxorubicin cardiomyopathic rat model, probucol prevents the development of doxorubicin-induced cardiomyopathy by increasing ATP production in cardiac tissues [24]. Moreover, in isoproterenol-induced rat model of heart failure, probucol was shown to attenuate oxidative stress and energy depletion [25]. Probucol attenuates the progression of congestive heart failure and cardiomyocyte apoptosis induced by ischemia reperfusion [26]. It protected rat heart against ischemia-reperfusion arrhythmias-induced cardiac damage [27]. Recently, it has been reported that other cholesterol lowering drugs such as simvastatin have been shown to be neuroprotective against cerebral ischemia-induced neuronal damage [28–31]. Although the therapeutic potential of probucol for the treatment of many forms of heart diseases, including myocardial ischemia reperfusion, is well documented, its direct role in forebrain ischemia-induced hippocampal damage has not been studied yet. Therefore, this study aimed to examine whether or not probucol would protect rat hippocampal neurons from death after forebrain ischemia.

## 2. Results

**Figure 1:**
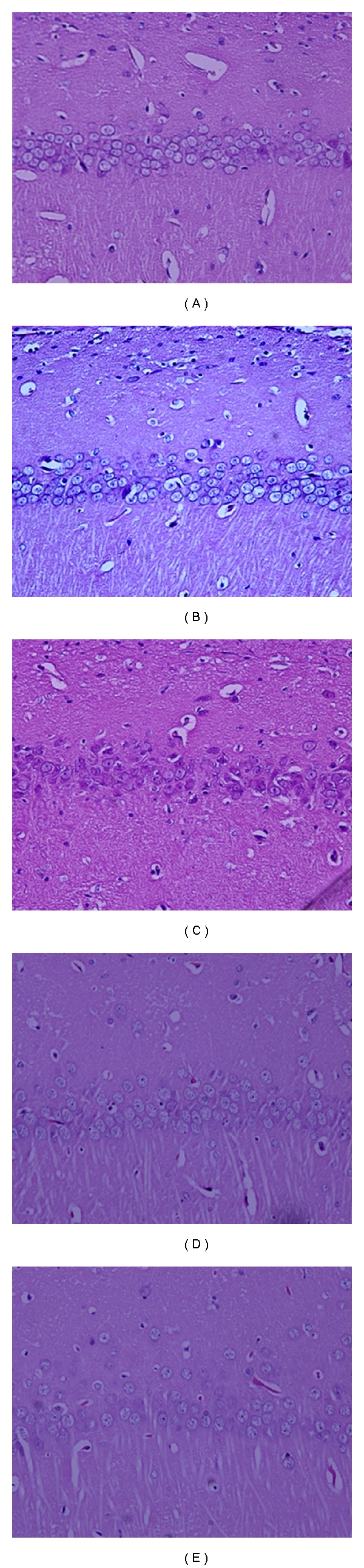
Effects of forebrain ischemia, probucol and their combination on the number of intact neurons in CA1 subregion of rat hippocampus. Hippocampal CA1 subregion from the control (a), sham-operated (b), and probucol-treated (d) groups showing normal intact neurons (×20). Hippocampal CA1 subregion from rats subjected to 10 min forebrain ischemia group (c) showed severe and marked decrease in the number of intact neurons (×20). Hippocampal CA1 subregion from rats treated with probucol plus ischemia group (e) showed normal intact neurons (×20).

The effects of 10 min of forebrain ischemia, probucol and their combination on the number of intact neurons in the CA1 region of rat hippocampus, are shown in Figure 1. Histopathological examination of the hippocampal CA1 region from the control (Figure 1(a)), sham-operated (Figure 1(b)), and probucol-treated (Figure 1(d)) groups revealed normal intact neurons. However, 7 days after the forebrain ischemia, there was widespread damage to the CA1 region of the hippocampus, as demonstrated by a highly significant 77% decrease in the number of intact neurons (Figure 1(c)). Neuronal death in the CA1 hippocampal sector was significantly reduced by the administration of probucol immediately after ischemia and continued for 7 successive days (Figure 1(e)). This was manifested as a significant increase in the number of intact neurons as compared to the ischemia group. 

**Figure 2:**
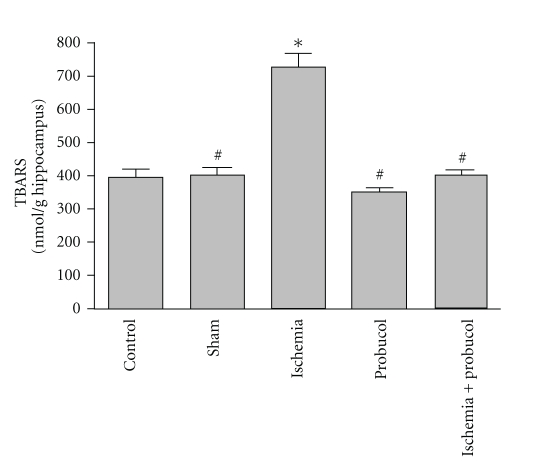
Effects of forebrain ischemia, probucol, and their combination on TBARS level, an index of lipid peroxidation, in hippocampal tissues in each group. Data are presented as mean ± S.E.M. (*n* = 10). * and ^#^ indicate significant change from control and ischemia, respectively, at *P* < 0.05 using ANOVA followed by Tukey-Kramer as a post-ANOVA test.

The effects of forebrain ischemia, probucol and their combination on TBARS level, an index of lipid peroxidation, in hippocampal tissues are shown in Figure 2. Forebrain ischemia induced a significant 71% increase in hippocampal TBARS level compared with both sham-operated and control groups. Treatment of ischemic rats with probucol resulted in complete reversal of the forebrain ischemia-induced increase in TBARS level to the control values. However, the administration of probucol alone for 7 successive days resulted in nonsignificant changes in hippocampal TBARS.

**Figure 3:**
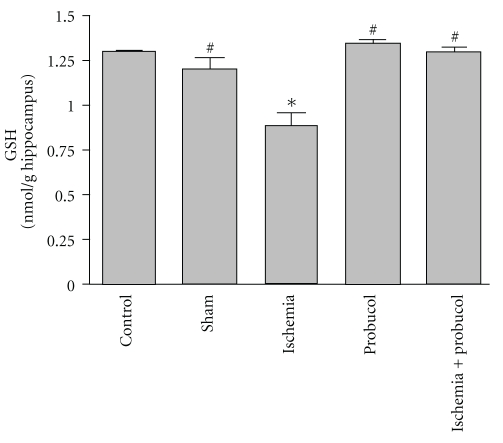
Effects of forebrain ischemia, probucol, and their combination on GSH level, an index antioxidant, in hippocampal tissues in each group. Data are presented as mean ± S.E.M. (*n* = 10). * and ^#^ indicate significant change from control and ischemia, respectively, at *P* < 0.05 using ANOVA followed by Tukey-Kramer as a post-ANOVA test.

Figure 3 shows the effects of probucol, ischemia, and their combination on GSH level, which reflects the cellular nonenzymatic antioxidant defense system in rat hippocampal tissues. Forebrain ischemia induced a significant 32% decrease in hippocampal GSH level. The administration of probucol immediately after ischemia and continued for 7 successive days resulted in a reversal of the forebrain ischemia-induced decrease in GSH level to the control values.

**Figure 4:**
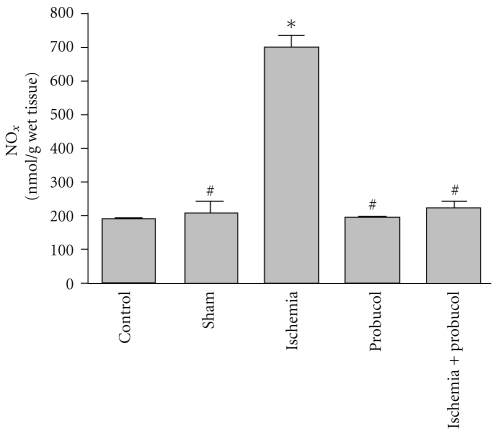
Effects of forebrain ischemia, probucol, and their combination on NO*_x_* concentrations, an index reactive nitrogen species, in hippocampal tissues in each group. Data are presented as mean ± S.E.M. (*n* = 10). * and ^#^ indicate significant change from control and ischemia, respectively, at *P* < 0.05 using ANOVA followed by Tukey-Kramer as a post-ANOVA test.

The effects of forebrain ischemia, probucol and their combination on NO*_x_* concentrations in rat hippocampal tissues, are shown in Figure 4. Forebrain ischemia induced a significant 260% increase in NO*_x_* concentration in hippocampal tissues. Treatment of ischemic rats with probucol resulted in a reversal of the forebrain ischemia-induced increase in NO*_x_* concentration to the control values. The administration of probucol alone for 7 successive days resulted in nonsignificant changes in hippocampal NO*_x_* concentration.

**Figure 5:**
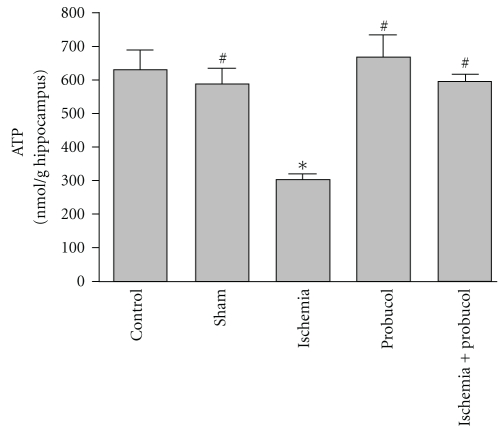
Effects of forebrain ischemia, probucol, and their combination on ATP concentrations, an index of energy production and mitochondrial function, in hippocampal tissues in each group. Data are presented as mean ± S.E.M. (*n* = 10). * and ^#^ indicate significant change from control and ischemia, respectively, at *P* < 0.05 using ANOVA followed by Tukey-Kramer as a post-ANOVA test.

Figure 5 shows the effects of 10 min forebrain ischemia, probucol and their combination on ATP level in the rat hippocampus. Forebrain ischemia resulted in a significant 51% decrease in hippocampal ATP level compared with the sham-operated and control groups. Administration of probucol (61 mg/kg) alone for 7 successive days resulted in a nonsignificant increase in hippocampal ATP level. However, treatment of ischemic rats with probucol immediately after ischemia and continued for 7 successive days resulted in complete reversal of the forebrain ischemia-induced depletion of ATP level in hippocampal tissues to the control values.

## 3. Discussion

In the present study, the administration of probucol to rats prevented delayed neuronal death of the hippocampal CA1 region induced by transient forebrain ischemia. This protection was evident from the significant reduction in neuronal cell death in the hippocampal CA1 region, restoration of the non-protein-SH contents and reduction in TBARS production, prevention of the elevation in NO*_x_* concentrations, and normalization of ATP level after forebrain ischemia. Additionally, the administration of probucol resulted in complete recovery of the decrease in ATP levels observed after ischemia compared to the control value. 

 Results of this study indicated that 10 min of forebrain ischemia in rats induced massive and selective neuronal damage in vulnerable regions, namely, the hippocampal CA1 region. About 77% of the hippocampal CA1 neurons died after 7 days postischemic reperfusion (Figure 1(c)). These results confirm those previously reported [3, 14, 15, 32]. The administration of probucol to ischemic rats offered protection against hippocampal CA1 neuronal damage induced by the 10 min of forebrain ischemia as evidenced by the fact that probucol rescued most of CA1 pyramidal neurons from ischemic death (Figure 1(e)).

In the present study, the concentration of ATP in rat hippocampus was measured 7 days after reperfusion and was found to be decreased by 51% of the control values (Figure 5). These results are consistent with those previously reported that in CA1 region of the hippocampus, a decrease in ATP and phosphocreatine occurs beyond 48 hours of reperfusion [7, 8]. Under similar experimental condition, previous studies by the author have reported that forebrain ischemia decreased ATP production in rat hippocampus 7 days after reperfusion [14, 15]. This observed decrease in ATP production in rat hippocampus after forebrain ischemia could be a secondary event following the inhibition of substrate utilization and mitochondrial oxidative phosphorylation. It has been reported that alterations in mitochondrial respiration can induce secondary depletion of both phosphocreatine and ATP [8], as well as increased levels of brain lactate and decreased activity of pyruvate dehydrogenase enzyme [33]. Furthermore, the accumulation of the toxic ROS following ischemia markedly increases the susceptibility of brain tissues to oxidative damage via membrane lipid peroxidation with the consequent decrease in the activity of many mitochondrial enzymes [34, 35]. 

Forebrain ischemia significantly increased lipid peroxidation in the hippocampus, which was measured by TBARS, as compared to levels in the normal hippocampus. These results are in agreement with earlier studies in our laboratory and others [11–15]. Lipid peroxidation, due to free radicals attacking cellular membranes, results in the formation of the lipid peroxidation byproducts including 4-hydroxynonenal, malondialdehyde, and many other toxic aldehydes which are highly reactive with TBA and toxic to neuronal perikarya, axons, and oligodendrocytes [36]. This study reports an increase in NO*_x_* concentrations after ischemia. Nitric oxide (NO) is a potent oxidizing molecule capable of eliciting lipid peroxidation and cellular damage [37]. It has been suggested that NO has the ability to exert multiple cytotoxic effects including an increase in arachidonic acid metabolism as well as the formation of peroxynitrite, ONOO^−^. Nitric oxide may also damage DNA through nucleotide base deamination and may trigger programmed cell death [38]. There was also a significant reduction in the level of glutathione in the ischemic hippocampal neurons. Glutathione, an endogenous antioxidant found in animal cells, reacts with radicals and provides protection from free radical damage. Both increased level of TBARS and reduced glutathione level in the ischemic hippocampus suggest an increased load of free radicals during ischemia-reperfusion injury. 

Typically, equilibrium exists between the generation of ROS and the antioxidant defenses, maintaining homeostatic control over the cell's oxidative state. Hippocampal neurons may be particularly susceptible to oxidative stress because of the high rate of oxidative metabolic activity and low level of antioxidant enzymes. Glutathione is considered to be the most important intracellular nonprotein thiol compound in mammalian cells and plays a crucial role as a free radical scavenger, particularly effective against the hydroxyl radical, for which there is no known enzymatic defense system. Therefore, the ability of glutathione to nonenzymatically scavenge both singlet oxygen and hydroxyl radical provides a first line of antioxidant defense [39]. There has been strong evidence that glutathione depletion causes nerve cell death after forebrain ischemia [14, 15]. The loss of glutathione may cause mitochondrial damage, and the impairment of mitochondrial function may lead to a decrease in cytosolic glutathione. In addition to its critical role as a free radical scavenger, glutathione may act as a redox modulator of ionotropic receptors and serve as a neuroprotectant against glutamate excitotoxicity, which together suggest that alterations in glutathione status may be deleterious to normal neuronal function [40, 41]. 

Since free radicals and energy depletion are increasingly implicated as key mediators of neuronal injury, neuroprotective antioxidants and ATP level boosters are considered a promising approach to limit the extent of neuronal cell loss. For instance, many antioxidants are reported to reduce ROS-mediated reactions and rescue hippocampal neurons from ischemia-reperfusion-induced neuronal loss in animal models of forebrain ischemia [14, 15, 35, 42, 43]. Additionally, statins protect against cerebral ischemia by limiting oxidative stress-induced damage [28–30].

In the present study, the administration of probucol to ischemic rats offered considerable protective effect manifested by the normalization of ATP level and increasing the number of intact neurons in hippocampus. This observed increase in ATP production by probucol was parallel to the increase in the number of intact neurons in hippocampus, which may point to the possible consideration that ATP could be a key mechanism by which probucol attenuates forebrain ischemia-induced neuronal damage. This hypothesis is consistent with previous studies which have reported that probucol increases ATP/ADP ratio which is essential for mitochondria function in myocardial ischemia reperfusion [25, 44]. A possible explanation for this effect is that probucol could improve substrate oxidation and mitochondrial oxidative phosphorylation secondary to its powerful antioxidant activity which preserves the activity of many mitochondrial enzymes. 

Probucol produced a significant reduction of ischemia-induced oxidative stress which may be attributed to at least partly to the restoration of the non-protein-SH contents and reduction in TBARS production. Probucol also significantly prevented the elevation in NO*_x_* concentrations. Probucol attenuated the loss of total glutathione caused by forebrain ischemia, which provided evidence that probucol treatment decreases oxidative stress by restoring reduced level of the natural antioxidant, glutathione. This may be achieved via its modulatory action on the altered glutathione metabolism. The antioxidant activities of probucol have been previously reported [18–21]. More recently, Asiri have reported that probucol not only increases the activity of endogenous antioxidant enzymes but also it increases the mRNA expression of antioxidant genes and inhibits apoptosis in cardiac tissues with the consequent improvement in mitochondrial oxidative phosphorylation and energy production [44].

## 4. Methods

### 4.1. Animals

A total of 75 adult male Wistar albino rats, weighing 230–250 g, were obtained from the Animal Care Center, College of Pharmacy, King Saud University, Riyadh, Saudi Arabia and were housed in metabolic cages under controlled environmental conditions (25°C and a 12 h light/dark cycle). Animals had free access to pulverized standard rat pellet food and tap water unless otherwise indicated. The protocol of this study has been approved by the Research Ethics Committee of the College of Pharmacy, King Saud University, Riyadh, Saudi Arabia.

### 4.2. Drugs and Chemicals

Probucol (Sigma Chemical Co., St. Louis, Mo, USA) was dissolved in corn oil and administered intraperitoneally (I.P) at a dose of 61 mg/kg according to previous studies [23, 25, 44]. Intraperitoneal injection was selected because probucol is poorly absorbed from the gastrointestinal tract, with only 2–8% of the dose reaching the circulation [45]. Thiobarbituric acid was purchased from Sigma Chemical Co. (St. Louis, Mo, USA), while Ellman's reagent (5-5′-dithiobis-2-nitrobenzoic acid; DTNB) was purchased from Fluka Chemical Company (Switzerland). All other chemicals were of the highest analytical grades commercially available.

### 4.3. Experimental Treatment Protocols

75 adult male Wistar albino rats were randomly divided into 5 groups of 15 animals each. Rats in the first group (control group) were injected with the vehicle of probucol, corn oil, (0.5 mL/200 g body weight/day, I.P.) for 7 successive days. Rats in the second group (sham group) were subjected to sham-operated ischemia and injected with the vehicle of probucol, corn oil, for 7 successive days. Rats in the third group (ischemia group) were injected with the same dose of corn oil immediately after the induction of 10 min forebrain ischemia and continued for 7 successive days. Rats in the fourth group (probucol group) were injected with probucol (61 mg/kg/day, I.P.) for 7 successive days. Rats in the fifth group (ischemia plus probucol group) were injected with the same dose of probucol immediately after the induction of 10 min forebrain ischemia and continued for 7 successive days.

### 4.4. Transient Forebrain Ischemia Model

Transient forebrain ischemia was induced in the rats under general anaesthesia (sodium pentobarbital; 30 mg/kg, I.P.) with 2-vessel occlusion combined with systemic hypotension according to the method of Smith et al. [46] and Henrich-Noack et al. [47]. First, blood was gradually withdrawn from jugular vein into a heparinized syringe to reduce systemic blood pressure to 45–50 mmHg. With the animal in supine, the common carotid arteries were exposed by means of a ventral midline neck incision. Both common carotid arteries were exposed, separated from the vagus nerve, and occluded for 10 minutes with microaneurysmal clips (Dieffenbach Bulldog Clamp, 25 mm, straight, Germany). At the end of the occlusion period, the clamps were released allowing the restoration of carotid blood flow, and the incision was sutured with 2-0 silk sutures. In sham-operated animals, the arteries were freed from connective tissue but were not occluded. Body temperature was kept at 37°C by using a controlled heating pad and heating lamps throughout the entire period of ischemia and post-ischemic recovery under anesthesia. A rectal thermometer was used to monitor body temperature (Apelex Rectal Thermometer, Panlab, Bagneux, France).

### 4.5. Histological Analysis of Hippocampal CA1 Region

Seven days after ischemia, 5 rats from each group were anesthetized with sodium pentobarbital (100 mg/kg). Rats were then transcardially perfused with cold saline followed by 4% formalin in phosphate-buffered saline (0.1 M; pH 7.4). The brains were removed from the skull and fixed in the same fixative for 24 h. Thereafter, the brains were embedded in paraffin, and 5 *μ*m thick sections were coronally cut at the level of the dorsal hippocampus by a rotatory microtome (Leica CM3050S, Leica Microsystems, Bensheim, Germany). The segments of the hippocampal CA1 region per 1000-*μ*m lengths from bregma −3.3, −3.8, and −4.3 were counted for viable cells. Tissue sections were stained with hematoxylin and eosin. The hippocampal damage was determined by counting the number of intact neurons in the stratum pyramidal within the CA1 subfield at a magnification of 20 (Nikkon E 600, digital camera DXM1200F, Nikon Corporation, Tokyo, Japan). Only neurons with normal visible nuclei were counted. The mean number of CA1 neurons per millimeter linear length for both hemispheres in sections of dorsal hippocampus was calculated for each group of animals. An observer who was unaware of the condition for each rat made all assessments of the histological sections.

### 4.6. Tissue Sampling

Seven days after ischemia, the remaining 10 rats from each group were decapitated, brains were quickly removed, and the hippocampi were harvested on a cold stage. Hippocampi were washed with saline, blotted dry on a filter paper, weighed and then 10% (w/v) homogenates were made in 6% perchloric acid (for assessment of ATP) and in ice-cold saline (for assessment of hippocampal tissue contents of GSH, TBARS, and NO*_x_*, using Branson Sonifier (VWR Scientific, Danburg, USA)

### 4.7. Assay of Lipid Peroxidation and Reduced Glutathione

The degree of lipid peroxidation in the hippocampal neuronal tissue was determined by measuring TBARS in the supernatant tissue from homogenate [48]. The homogenates were centrifuged at 3500 rpm, and supernatant was collected and used for the estimation of TBARS. The absorbance was measured spectrophotometrically at 532 nm, and the concentrations were expressed as nmol TBARS/g wet tissue. The tissue levels of the acid soluble thiols, GSH, were assayed calorimetrically at 412 nm according to the method of Ellman [49], using a Shimadzu (Tokyo, Japan) spectrophotometer. The contents of GSH were expressed as *μ*mol/g wet tissue [14, 15].

### 4.8. Determination of Total Nitrate/Nitrite (NO_x_) Concentrations in Hippocampal Tissue

Total nitrate/nitrite (NO*_x_*) concentrations were measured as stable end product, nitrite, according to the method of Miranda et al. [50]. The assay is based on the reduction of nitrate by vanadium trichloride combined with detection by the acidic griess reaction. The diazotization of sulfanilic acid with nitrite at acidic pH and subsequent coupling with N-(10 naphthyl)-ethylenediamine produced an intensely colored product that is measured spectrophotometrically at 540 nm. The concentrations of NO*_x_* were expressed as nmol/g wet tissue [14, 15].

### 4.9. Determination of Adenosine Triphosphate Production in Hippocampal Tissue

Adenosine triphosphate was determined in hippocampal tissue using HPLC according to the method of Botker et al. [51]. In brief, hippocampal tissue was homogenized in ice-cold 6% perchloric acid, centrifuged at 1000 rpm for 15 min at 0.5°C, and the supernatant fluid was injected into HPLC after neutralization to pH 6-7. Chromatographic separation was performed at a flow rate of 1.2 mL/min, using ODS-Hypersil, 150 × 4.6 mm I.D., 5 *μ*m column (Supelco SA, Gland, Switzerland) and 75 mM ammonium dihydrogen phosphate as mobile phase. The peak elution was followed at 254 nm. The concentrations of ATP were expressed as nmol ATP/g wet tissue [14, 15].

### 4.10. Statistical Analysis

Differences between obtained values (mean ± S.E.M.) were carried out by one way analysis of variance (ANOVA) followed by the Tukey-Kramer multiple comparison test. A *P* value of 0.05 or less was taken as a criterion for a statistically significant difference.

## 5. Conclusions

Data from this study suggest that the administration of probucol protected rats from ischemia-induced brain injury. This protection may be due to the reduction of oxidative stress and boosting of hippocampal ATP levels. These observations suggest that probucol may be a clinically viable protective agent against a variety of conditions where cellular damage is a consequence of oxidative stress and energy depletion. In addition, probucol may have the potential to be used in the prevention of neurodegenerative diseases such as forebrain ischemia. However, other clinical considerations must also be taken into account, such as the diminishing effect of probucol on both LDL and HDL content. Nevertheless, the results of this study open new perspectives for the use of probucol in the treatment of neurodegenerative diseases particularly those that are associated with or secondary to myocardial ischemia.

##  Conflict of Interest

The author of this study declare that there is no conflict of interest.

## References

[1] Levy DE, Caronna JJ, Singer BH, Lapinski RH, Frydman H, Plum F (1985). Predicting outcome from hypoxic-ischemic coma. *Journal of the American Medical Association*.

[2] Petito CK, Feldmann E, Pulsinelli WA, Plum F (1987). Delayed hippocampal damage in humans following cardiorespiratory arrest. *Neurology*.

[3] Kirino T (1982). Delayed neuronal death in the gerbil hippocampus following ischemia. *Brain Research*.

[4] Pulsinelli WA, Brierley JB, Plum F (1982). Temporal profile of neuronal damage in a model of transient forebrain ischemia. *Annals of Neurology*.

[5] Kirino T (2000). Delayed neuronal death. *Neuropathology*.

[6] Colbourne F, Li H, Buchan AM (1999). Continuing postischemic neuronal death in CA1: influence of ischemia duration and cytoprotective doses of NBQX and SNX-111 in rats. *Stroke*.

[7] Sims NR, Zaidan E (1995). Biochemical changes associated with selective neuronal death following short-term cerebral ischaemia. *International Journal of Biochemistry and Cell Biology*.

[8] Arai H, Passonneau JV, Lust WD (1986). Energy metabolism in delayed neuronal death of CA1 neurons of the hippocampus following transient ischemia in the gerbil. *Metabolic Brain Disease*.

[9] Katsura K, Kristián T, Smith M-L, Siesjö BK (1994). Acidosis induced by hypercapnia exaggerates ischemic brain damage. *Journal of Cerebral Blood Flow & Metabolism*.

[10] Iadecola C, Pelligrino DA, Moskowitz MA, Lassen NA (1994). Nitric oxide synthase inhibition and cerebrovascular regulation. *Journal of Cerebral Blood Flow & Metabolism*.

[11] Chan PH (2001). Reactive oxygen radicals in signaling and damage in the ischemic brain. *Journal of Cerebral Blood Flow & Metabolism*.

[12] Evans PH (1993). Free radicals in brain metabolism and pathology. *British Medical Bulletin*.

[13] Al-Majed AA, Al-Yahya AA, Asiri Y, Al-Gonaiah MA, Mostafa AM (2004). Nimesulide prevents oxidative stress damage following transient forebrain ischemia in the rat hippocampus. *Research Communications in Molecular Pathology and Pharmacology*.

[14] Al-Majed AA, Sayed-Ahmed MM, Al-Omar FA, Al-Yahya AA, Aleisa AM, Al-Shabanah OA (2006). Carnitine esters prevent oxidative stress damage and energy depletion following transient forebrain ischaemia in the rat hippocampus. *Clinical and Experimental Pharmacology and Physiology*.

[15] Al-Majed AA, Al-Omar FA, Nagi MN (2006). Neuroprotective effects of thymoquinone against transient forebrain ischemia in the rat hippocampus. *European Journal of Pharmacology*.

[16] Gupta A, Bhatt ML, Misra MK (2009). Lipid peroxidation and antioxidant status in head and neck squamous cell carcinoma patients. *Oxidative Medicine and Cellular Longevity*.

[17] Fisher-Wellman K, Bell HK, Bloomer RJ (2009). Oxidative stress and antioxidant defense mechanisms linked to exercise during cardiopulmonary and metabolic disorders. *Oxidative Medicine and Cellular Longevity*.

[18] Zimetbaum P, Eder H, Frishman W (1990). Probucol: pharmacology and clinical application. *Journal of Clinical Pharmacology*.

[19] Kaul N, Siveski-Iliskovic N, Hill M, Khaper N, Seneviratne C, Singal PK (1996). Probucol treatment reverses antioxidant and functional deficit in diabetic cardiomyopathy. *Molecular and Cellular Biochemistry*.

[20] Siveski-Iliskovic N, Hill M, Chow D, Singal PK (1995). Probucol protects against adriamycin cardiomyopathy without interfering with its antitumor effect. *Circulation*.

[21] Li T, Singal PK (2000). Adriamycin-induced early changes in myocardial antioxidant enzymes and their modulation by probucol. *Circulation*.

[22] Sia YT, Parker TG, Liu P, Tsoporis JN, Adam A, Rouleau JL (2002). Improved post-myocardial infarction survival with probucol in rats: effects on left ventricular function, morphology, cardiac oxidative stress and cytokine expression. *Journal of the American College of Cardiology*.

[23] Sia YT, Lapointe N, Parker TG (2002). Beneficial effects of long-term use of the antioxidant probucol in heart failure in the rat. *Circulation*.

[24] El-Demerdash E, Awad AS, Ali AA, Sayed-Ahmed MM, Osman AM (2003). New aspects in probucol cardioprotection against doxorubicin-induced cardiotoxicity. *Cancer Chemotherapy and Pharmacology*.

[25] El-Demerdash E, Awad AS, Taha RM, El-Hady AM, Sayed-Ahmed MM (2005). Probucol attenuates oxidative stress and energy decline in isoproterenol-induced heart failure in rat. *Pharmacological Research*.

[26] Ruixing Y, Al-Ghazali R, Wenwu L, Jinzhen W (2006). Pretreatment with probucol attenuates cardiomyocyte apoptosis in a rabbit model of ischemia/reperfusion. *Scandinavian Journal of Clinical and Laboratory Investigation*.

[27] Tada H, Oida K, Kutsumi Y, Shimada Y, Nakai T, Miyabo S (1992). Effects of probucol on impaired cardiac performance and lipid metabolism in streptozotocin-induced diabetic rats. *Journal of Cardiovascular Pharmacology*.

[28] Beretta S, Pastori C, Sala G (2011). Acute lipophilicity-dependent effect of intravascular simvastatin in the early phase of focal cerebral ischemia. *Neuropharmacology*.

[29] Guluma KZ, Lapchak PA (2010). Comparison of the post-embolization effects of tissue-plasminogen activator and simvastatin on neurological outcome in a clinically relevant rat model of acute ischemic stroke. *Brain Research*.

[30] Lampl Y, Lorberboym M, Gilad R (2010). Early outcome of acute ischemic stroke in hyperlipidemic patients under atorvastatin versus simvastatin. *Clinical Neuropharmacology*.

[31] Lapchak PA, Han MK (2010). Simvastatin improves clinical scores in a rabbit multiple infarct ischemic stroke model: synergism with a ROCK inhibitor but not the thrombolytic tissue plasminogen activator. *Brain Research*.

[32] Knuckey NW, Palm D, Primiano M, Epstein MH, Johanson CE (1995). N-acetylcysteine enhances hippocampal neuronal survival after transient forebrain ischemia in rats. *Stroke*.

[33] Katayama Y, Welsh FA (1989). Effect of dichloroacetate on regional energy metabolites and pyruvate dehydrogenase activity during ischemia and reperfusion in gerbil brain. *Journal of Neurochemistry*.

[34] Al Nita D, Nita V, Spulber S (2001). Oxidative damage following cerebral ischemia depends on reperfusion—a biochemical study in rat. *Journal of Cellular and Molecular Medicine*.

[35] Al-Majed AA (2004). Aminoguanidine prevents oxidative stress insult following transient forebrain ischemia in the rat hippocampus. *Saudi Pharmaceutical Journal*.

[36] McCracken E, Valeriani V, Simpson C, Jover T, McCulloch J, Dewar D (2000). The lipid peroxidation by-product 4-hydroxynonenal is toxic to axons and oligodendrocytes. *Journal of Cerebral Blood Flow & Metabolism*.

[37] Beckman JS, Beckman TW, Chen J, Marshall PA, Freeman BA (1990). Apparent hydroxyl radical production by peroxynitrite: implications for endothelial injury from nitric oxide and superoxide. *Proceedings of the National Academy of Sciences of the United States of America*.

[38] Iadecola C (1997). Bright and dark sides of nitric oxide in ischemic brain injury. *Trends in Neurosciences*.

[39] Coyle JT, Puttfarcken P (1993). Oxidative stress, glutamate, and neurodegenerative disorders. *Science*.

[40] Mithöfer K, Sandy MS, Smith MT, Di Monte D (1992). Mitochondrial poisons cause depletion of reduced glutathione in isolated hepatocytes. *Archives of Biochemistry and Biophysics*.

[41] Bains JS, Shaw CA (1997). Neurodegenerative disorders in humans: the role of glutathione in oxidative stress-mediated neuronal death. *Brain Research Reviews*.

[42] Aureli T, Miccheli A, Di Cocco ME (1994). Effect of acetyl-l-carnitine on recovery of brain phosphorus metabolites and lactic acid level during reperfusion after cerebral ischemia in the rat—study by ^13^P- and ^1^H-NMR spectroscopy. *Brain Research*.

[43] Calvani M, Arrigoni-Martelli E (1999). Attenuation by acetyl-L-carnitine of neurological damage and biochemical derangement following brain ischemia and reperfusion. *International Journal of Tissue Reactions*.

[44] Asiri YA (2010). Probucol attenuates cyclophosphamide-induced oxidative apoptosis, p53 and Bax signal expression in rat cardiac tissues. *Oxidative Medicine and Cellular Longevity*.

[45] Yamamoto K, Fukuda N, Shiroi S (1994). Effects of dietary fat levels on the absorption and tissue accumulation of probucol in the rat. *Arzneimittel-Forschung*.

[46] Smith ML, Bendek G, Dahlgren N (1984). Models for studying long term recovery following forebrain ischemia in the rat, II: a 2-vessel occlusion model. *Acta Neurologica Scandinavica*.

[47] Henrich-Noack P, Prehn JHM, Krieglstein J (1996). TGF-*β*1 protects hippocampal neurons against degeneration caused by transient global ischemia: dose-response relationship and potential neuroprotective mechanisms. *Stroke*.

[48] Ohkawa H, Ohishi N, Yagi K (1979). Assay for lipid peroxides in animal tissues by thiobarbituric acid reaction. *Analytical Biochemistry*.

[49] Ellman GL (1959). Tissue sulfahydryl groups. *Archives of Biochemistry and Biophysics*.

[50] Miranda KM, Espey MG, Wink DA (2001). A rapid, simple spectrophotometric method for simultaneous detection of nitrate and nitrite. *Nitric Oxide: Biology and Chemistry*.

[51] Botker HE, Kimose M, Helligso P, Nielsen TT (1994). Analytical evaluation of high energy phosphate determination by high performance liquid chromatography in myocardial tissue. *Journal of Molecular and Cellular Cardiology*.

